# Representative Seroprevalences of Human and Livestock Brucellosis in Two Mongolian Provinces

**DOI:** 10.1007/s10393-014-0962-7

**Published:** 2014-07-11

**Authors:** Baljinnyam Zolzaya, Tsend Selenge, Tsegeen Narangarav, Dorj Gantsetseg, Dashzevge Erdenechimeg, Jakob Zinsstag, Esther Schelling

**Affiliations:** 1 Swiss Tropical and Public Health Institute, P.O. Box CH 4002 Basel, Switzerland; 2 Animal Health Project, Swiss Agency for Development and Cooperation SDC, Government Building 11, Room 601, J.Sambuu street -11, Chingeltei District 4, Ulaanbaatar, 15141 Mongolia; 3 University of Basel, Basel, Switzerland; 4 National Centre for Communicable Diseases, Ulaanbaatar, Mongolia; 5 State Central Veterinary Laboratory, Zaisan, Ulaanbaatar, Mongolia; 6 Veterinary Research Institute, Zaisan, Ulaanbaatar, Mongolia

**Keywords:** apparent seroprevalence, incidence, brucellosis, Mongolia, human, livestock

## Abstract

Mongolia implemented a brucellosis livestock mass vaccination campaign from 2000 to 2009. However, the number of human cases did not decline since 2004 and the current epidemiological situation in Mongolia was uncertain. The objective of this study was to estimate the representative seroprevalences of humans and livestock in two provinces in view of their comparison with officially reported data. A representative cross-sectional study using cluster sampling proportional to size in humans, sheep, goats, cattle, yaks, horses, camels and dogs was undertaken to assess the apparent seroprevalence in humans and animals. A total of 8054 livestock and dog sera and 574 human sera were collected in Sukhbaatar and Zavkhan provinces. Human and animal sera were tested with the Rose Bengal and ELISA tests. The overall apparent seroprevalence of brucellosis was 27.3% in humans (95% CI 23.7–31.2%), 6.2% (95% CI 5.5–7.1%) in sheep, 5.2% (95% CI 4.4–5.9%) in goats, 16.0% (95% CI 13.7–18.7%) in cattle, 2.5% (95% CI 0.8–7.6%) in camels, 8.3 (95% CI 6.0–11.6%) in horses and 36.4% (95% CI 26.3–48.0%) in dogs. More women than men were seropositive (OR = 1.7; *P* < 0.0014). Human seroprevalence was not associated with small ruminant and cattle seroprevalence at the nomadic camp (*hot ail*) level. Annual incidence of clinical brucellosis, inferred from the seroprevalence using a catalytic model, was by a factor of 4.6 (1307/280) in Sukhbaatar and by a factor of 59 (1188/20) in Zavkhan. This represents a 15-fold underreporting of human brucellosis in Mongolia. The lack of access to brucellosis diagnostic testing at the village level hinders rural people from receiving appropriate treatment. In conclusion, this study confirms the high seroprevalence of human and livestock brucellosis in Mongolia. Stringent monitoring and quality control of operational management of a nationwide mass vaccination of small and large ruminants is warranted to assure its effectiveness. More research is needed to understand the complex animal–human interface of brucellosis transmission at different scales from farm to provincial level.

## Introduction

Brucellosis is one of the most common zoonotic diseases worldwide. Brucellosis is endemic in livestock and causes human disease in Africa, Central America, Central Asia, the Mediterranean region and the Near East (Zinsstag et al. 2011; Bonfoh et al. 2012; Dean et al. 2012b). Brucellosis is caused by members of the *Brucella* genus. Infection is caused by *B. melitensis* in small ruminants and *B. abortus* in cattle and yaks. Brucellosis is mainly transmitted to humans through direct contact with infected livestock, placenta material and vaginal discharge or through the consumption of contaminated unpasteurised milk and dairy products. Human brucellosis causes varied clinical manifestations, including intermittent fever, sweating, joint and low back pain, headaches, fatigue, weight loss and general weakness persisting for a long time (Dean et al. 2012a).

Clinical signs of human brucellosis are often unspecific which makes it difficult to differentiate from other febrile conditions (Baba et al. 2001). Laboratory tests are essential for diagnosis and only the isolation of bacteria provides proof of infection. However, bacteriological diagnosis is expensive, difficult and dangerous. Serological tests are easier to implement in limited resource settings. In the absence of specific antibiotic treatment, brucellosis in humans may persist and progress to neurological or other severe complications (Diaz et al. 2011).

In Mongolia, traditional nomadic livestock systems are maintained where sheep, goats, cattle, yaks, camels and horses are kept in flocks of varying sizes. Herds move continuously depending on the availability of pasture during the summer and autumn, with limited mobility during the cold season. Herder families keep guard dogs to protect their livestock from wild predators. These dogs consume infected placenta/foetal materials and carcasses from the herds. Herders utilise traditional practices of home slaughtering animals for household consumption, milking mares to produce a traditional fermented drink called ‘airag’ and milking female camels to consume milk during the summer and autumn. Fresh cow’s milk is also used to feed weak newborn animals, which are brought into their traditional mobile houses during cold season (Madkour 2001).

In Mongolia, animal brucellosis was first documented in 1935 and human brucellosis was officially registered for the first time in 1949. The national livestock survey results showed that apparent brucellosis seroprevalences were 17% in cattle, 3.5% in sheep and 2% in goats in the 1950s. The government of Mongolia received assistance from World Health Organization to implement brucellosis control in the 1960s. A total of 4816 human sera were analysed during the national brucellosis survey, using the serum agglutination test (SAT) and the complement fixation test (CFT) to show that 1.7% of urban and 4.4% of rural populations were seropositive. Participants were classified according to occupational exposure risk as meat factory workers, sheep herders, dairy farmers and students from the Agricultural University. The national survey results provided evidence that human brucellosis had emerged in the country (Jezek et al. 1972).

The government of Mongolia was alarmed that human brucellosis had emerged in the country and urgently sought assistance from the Council for Mutual Economic Assistance (COMECON), the former economic assistance organisation of the Soviet Union. Subsequently, the government launched a national brucellosis control programme using a test-slaughter strategy from 1965 to 1968 (Enkhbaatar et al. 2004). In total, 37.5 million livestock and dogs were tested with an allergic skin test, CFT and SAT during the programme. The animals with positive serological test results were immediately separated from the herd and slaughtered within a short period of time (Kolar 1984). Mongolia was able to implement the test-and-slaughter strategy because the government owned all livestock. Therefore, it was not necessary to compensate herders when animals were slaughtered.

However, the government realised that the test-and-slaughter strategy was not practical and was less effective to control brucellosis under pastoralist conditions (Kolar 1987). An epidemiological team commissioned by WHO, conducted a Rev.1 vaccine trial for small ruminants, showing that the vaccine was highly effective for the local breeds of livestock in pastoralist conditions (Kolar 1982; Enkhbaatar et al. 2004) The Ministry of Agriculture approved a mass vaccination strategy using Rev. 1 vaccine for small ruminants and S19 vaccine for cattle and yaks, which was implemented from 1975 to 1985. More than 33 million small ruminants and 9.5 million cattle/yaks were vaccinated during the mass vaccination campaign, making it one of the most successful nationwide vaccination programmes in the world (Kolar 1987). The annual incidence of human brucellosis decreased from 25 per 100,000 in 1975 to 0.23 per 100,000 in 1986. However, the coverage achieved by mass vaccination campaigns was not assessed in a formal way (Kolar 1977; Selenge et al. 2011).

After 1990, Mongolia was no longer dependent on the former Soviet Union, and it went through democratic reform and privatisation of all sectors. The health care and veterinary services were weakened due to lack of resources during the post-socialist transition period. This influenced the breakdown of government-operated disease surveillance and control activities from 1990 onwards. Brucellosis re-emerged as a preventable human disease in most countries which were part of the former Soviet Union and Mongolia. Government reported human brucellosis annual incidence rose from 4.9 per 100,000 in 1990 to 67 per 100,000 in 1999 (Selenge et al. 2011). In a mathematical transmission model, a simultaneous fit of sheep-human and cattle-human contact rates indicated that >90% of the human brucellosis cases were related to small ruminant transmission (Zinsstag et al. 2005). However it was not known what proportion of the nomadic herders had access to brucellosis diagnosis and treatment in rural areas. It is possible that the true incidence was underestimated (Ebright et al. 2003). International experts recommended to the WHO that Mongolia needed to implement livestock brucellosis mass vaccination. In the year 2000, the Ministry of Food and Agriculture (MoFA) budgeted equivalent to 10.5 million USD for a 10-year livestock mass vaccination campaign, following the WHO recommendations. A cross-sector economic analysis showed the mass vaccination would be largely profitable to the Mongolian society (with a benefit-cost ratio of 3.2). If costs were shared between the public health sector and the agricultural sectors, cost-effectiveness could be at 20 USD per averted Disability-Adjusted Life Year (DALY) (Roth et al. 2003). However, the mass livestock vaccination campaign which started in 2000 seemed to lose traction as reported cases of human brucellosis did not decline further after 2004 (Selenge et al. 2011). The epidemiology of brucellosis in Mongolia became uncertain and needed to be reassessed. The objective of this study was to assess the seroprevalence of brucellosis in humans, different livestock species and dogs from two selected province (*aimags*). Additionally, the study aimed to evaluate the association between human and livestock seropositivity (Bonfoh et al. 2012). We hypothesised that human brucellosis seroprevalence could be statistically associated to brucellosis seroprevalence in small ruminants. The study results were compared with government reports to inform Mongolian policy makers from the medical and veterinary sectors to define the new strategy for the National Brucellosis Elimination program.

## Materials and Methods

### Study Design

This study took place from June to October, 2010. Human and livestock aggregated data was available at the provincial and national level from the Mongolian National Statistical Office. Household census information and official livestock annual census data from year 2009 was available from the district (*soum*) governors’ offices in all selected districts. A cross-sectional study design, similar to Bonfoh et al. (2012), utilised cluster sampling proportional to size for all species (Bennett 1991). An intraclass correlation coefficient (rho) of 0.1 between clusters was assumed, which is appropriate for highly contagious diseases like brucellosis (Otte and Gumm 1997). The sample size calculation was optimised to assure that the lower 95% confidence limit was higher than zero, and for larger prevalences, the largest standard error was 2.5%. The sample size was further optimised to assure the feasibility of sampling herds within the available budget. We assumed a brucellosis seroprevalence for livestock based on the reports of annual serological test results of Sukhbaatar and Zavkhan provinces from 1990 to 2008. Brucellosis seroprevalences were 0.5% for small ruminants, 2% for cattle and 3% for camels. Human brucellosis seroprevalence among rural populations was assumed to be 20% according to the Mongolian National Centre for Communicable Diseases (NCCD) (Davgadorj et al. 2003).

**Map 1:**
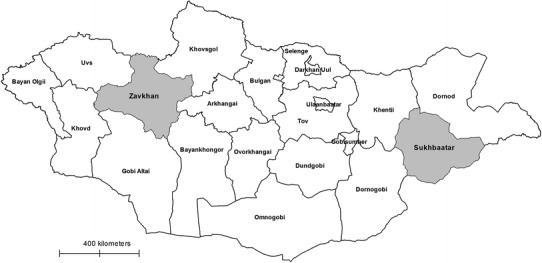
Mongolian map: selected Sukhbaatar and Zavkhan provinces are in *grey shade*

**Map 2:**
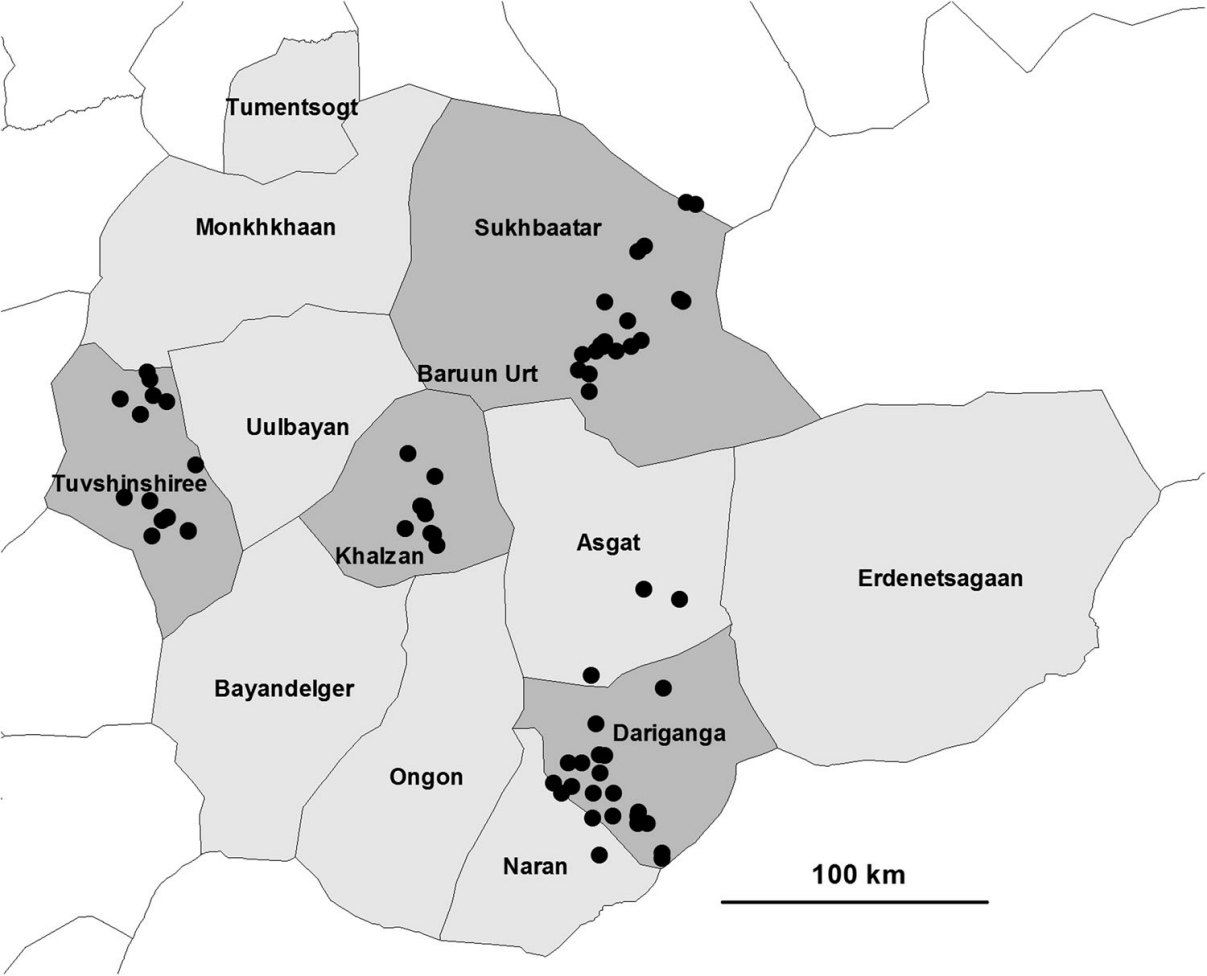
Location of participating nomadic camps from selected Dariganga, Khalzan, Sukhbaatar and Tuvshinshiree districts in *grey shade* from Sukhbaatar province

**Map 3:**
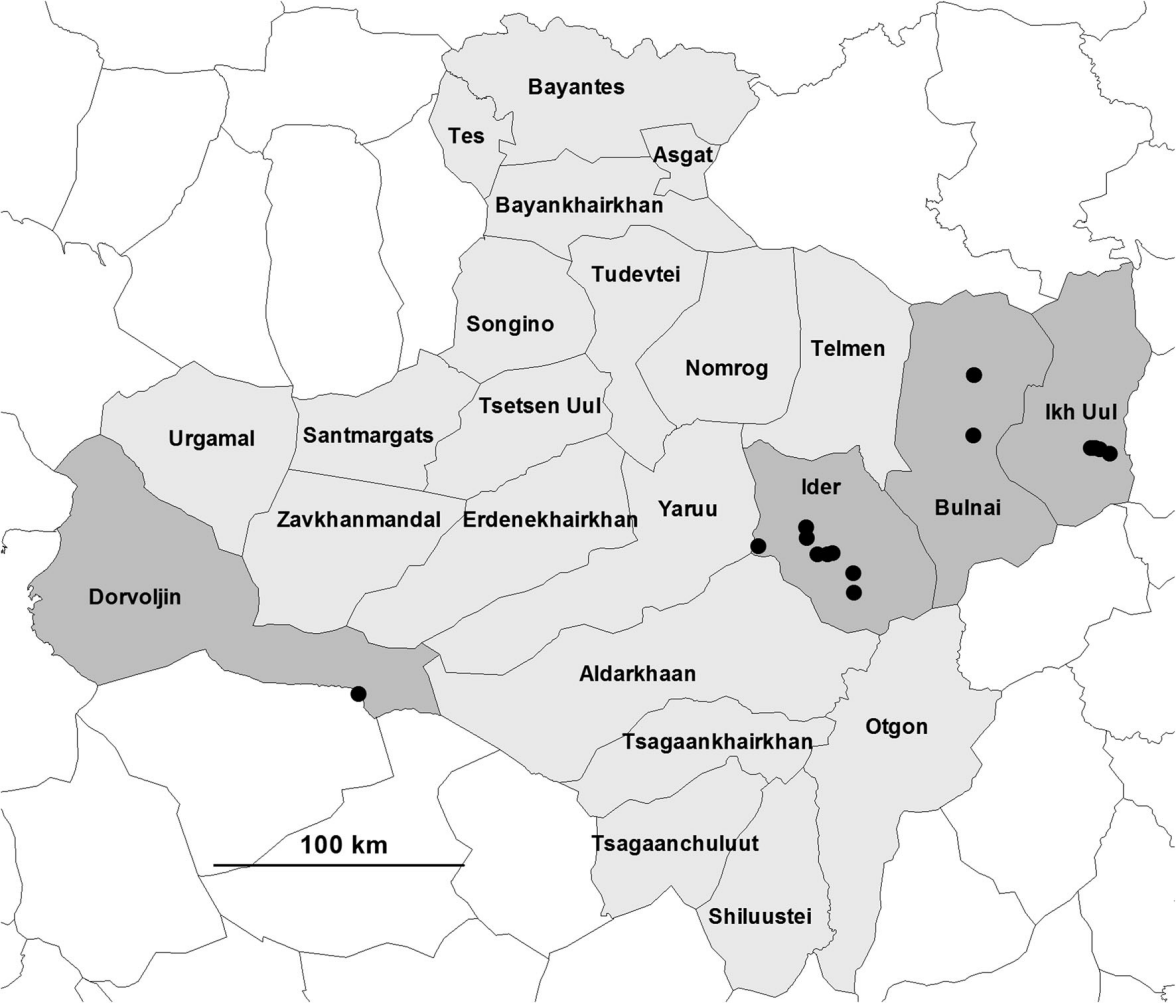
Location of participating nomadic camps from selected Durvuljin, Ider, Ikh-Uul and Tosontsengel districts in *grey shade* from Zavkhan province (only 17 of the 83 hot ails have the coordinates recorded)

Sukhbaatar and Zavkhan provinces were selected by the MoFA (Map 1). Zavkhan province represented the western region and Sukhbaatar province represented the eastern region of the country. Sukhbaatar province (Map 2) reported high human brucellosis incidence in the country annually and high livestock brucellosis prevalence, as well as high cross-province livestock movements. Human annual brucellosis incidence was not known, and sporadic cases were reported by Zavkhan province’s health department. There was low cross-province livestock movement in Zavkhan (Map 3).

Four districts were randomly selected proportional to size for livestock from each province. Similarly, the third level of sampling, 8 villages (*bags*), and the fourth stage, 10 nomadic camps (*hot ail*), were also randomly selected proportional to size. Nomadic camps were composed of two or three nomadic herder families camping together. Their herds of all different livestock species mix and shared the same pasture and water points. The selection of nomadic camps was done using official household lists of livestock annual census data from the governor’s office in eight villages (Bennett 1991). The nomadic camps were selected by drawing random numbers.

### Simultaneous Sampling of Human and Livestock

The study was conducted in the summer of 2010 by two field teams composed of three veterinarians, a medical doctor and a nurse. The field teams visited 86 nomadic camps from 4 districts of Sukhbaatar and 83 nomadic camps from 4 districts of Zavkhan province. The field team was able to find selected nomadic camps with the assistance of a local veterinarian. Approval was first sought from the chief of a nomadic camp and members were then gathered together to explain the aims and methods of study. Children under 16 years old were sampled if their parent or guardian provided written consent to participate in the study. Participants were selected from those who were willing by randomly drawing names from a hat. The planned sample size was 3–4 people per nomadic camp, thus totalling 60–80 people per district. A medical doctor collected blood samples from the participants. Basic data relating to demography (age, sex, birthdate), risk factors of exposure to infected livestock, consumption of dairy and animal source products and the presence of specific symptoms during the previous month (fever, headache, sweats, sleeping difficulties, fatigue, weight loss, joint and muscle pain or back pain) was collected through structured interviews with the participant or their guardian. Heads of nomadic camps were interviewed using structured questionnaires on livestock health status, number of aborted animals during last lambing season and brucellosis vaccination status of the herd for last 3 years. In each nomadic camp, 20 sheep, 20 goats, 5 cattle/yaks, 5 camels, 4 horses and a dog were selected and sampled if present during the field visit. Animals under 9 months of age and sick animals were excluded. Small ruminants were gathered in the sheep pen. A team member pointed at the first animal. From there every tenth small ruminant was selected until a total of 20 goats and 20 sheep were sampled (Otte and Gumm 1997). The selection of five cattle was made with a focus on cows. The team member pointed at the first horse and from there every second animal was selected until four horses were sampled. Any camel present at the time of the visit was sampled. Any dog which the owner was willing to hold was sampled.

### Serological Testing (RBT and ELISA)

Human and livestock blood samples were centrifuged the same day. Human serum samples were transported frozen in cool boxes, according to national biosafety standards, from the field site to the National Centre for Communicable Diseases (NCCD) in Ulaanbaatar. Livestock serum samples were transported in cool boxes from the field site to the State Central Veterinary Laboratory (SCVL) in Ulaanbaatar.

All human sera were tested by standard Rose Bengal Test (RBT; Tulip Diagnostics Ltd, Bambolim, India), mixing 25 µl antigen with 25 µl serum, which was read after 4 min. Positive sera were serially diluted with saline to obtain dilutions from 1/2 to 1/32. They were retested by the modified Rose Bengal Test protocol of Diaz et al. (2011). Additionally, all sera were tested by indirect IgG ELISA (Diagnostic Automation INC, California, USA). Twenty were positive by the standard RBT and modified RBT but negative by IgG ELISA. Those sera were retested by IgM ELISA (NovaLisa™, Nova Tec Immundiagnostica GmbH, Dietzenbach, Germany).

Study participants were asked about the following human risk factors: district where the family was staying at time of the study, age class, sex, occupation, consumption of raw milk and raw milk products, consumption of dried meat and raw or half-cooked liver. Husbandry practices recorded were: Keeping weak newborn animals in the family dwelling place, direct contact with livestock placentas/aborted foetuses and the disposal of aborted foetuses/placentas (feeding to dogs or discarding in the field). The observations of abortions among livestock species were also recorded.

Livestock sera were tested following the standard protocol of RBT (Biocombinate State Owned Enterprise, Ulaanbaatar, Mongolia and Brucellosis National Reference Center, Santa Fe, Spain). Cattle and yak sera were tested using 25 µl antigen and 25 µl serum ratio and small ruminant sera were tested with 25 µl antigen and 75 µl serum which was read after 4 min according to the guidelines of World Organization for Animal Health (OIE 2009). Camel, horse and dog sera were tested using 25 µl antigen and 25 µl serum according to the national standard. Additionally, livestock sera were tested by two competitive ruminant IgG ELISAs (COMPELISA of VLA^®^, UK and SVANOVIR^®^, Sweden).

### Data Analysis

Human and livestock data were double entered into an Access 2003 database (Microsoft, USA). Databases were validated using the ‘Data compare’ routine in Epi Info 3.5.1 (Centers for Disease Control and Prevention, USA). Human and small ruminants modified RBT results were compared with i-ELISA and c-ELISA. We report here apparent seroprevalence because the sensitivity (Se) and specificity (Sp) of RBT test for camels, yaks, horses and dogs were not established. True prevalences were estimated for some species using the Rogan–Gladen estimator TP = (AP + Sp − 1)/(Se + Sp − 1), where TP is the true prevalence, AP is the apparent prevalence, Se is the sensitivity and Sp is the specificity of Rose Bengal Test (Gladen and Rogan 1978). We considered that Se = 91.7% and Sp = 91.2% for human RBT produced by Tulip Diagnostics Ltd (Ruiz-Mesa et al. 2005). For small ruminants, we considered Se = 94% and Sp = 100 and for cattle, we used Se = 99.7 and Sp = 99% (Mainar-Jaime et al. 2005). The apparent seroprevalence was calculated considering clustering within nomadic camps.

The apparent human seroprevalence of the RBT was used to estimate the incidence of brucellosis exposure by a catalytic two way model under equilibrium conditions (Muench 1959)

1 $$ {\text{d}}S/{\text{d}}t = - aS + bI, $$

2 $$ {\text{d}}I/{\text{d}}t = aS - bI. $$

*S* is the susceptible population and *I* is the seropositive population. The parameter *a* is the incidence of seroconversion and *b* the rate of loss of sero-positivity. Under equilibrium conditions, the apparent seroprevalence *P* is related to *a* and *b* (3).

3 $$ P = a \, /\left( {a + b} \right). $$

We estimated the seroconversion rate *a* and loss of *b* simultaneously from the data using Microsoft Excel Software (Microsoft, USA)

The assumed average duration of brucellosis seropositivity (1/*b*) was 10.9 years (Bonfoh et al. 2012). Serological test results were converted into binary outcomes (0 = seronegative, 1 = seropositive), depending on the cut-off value of each test. Logistic regression, modelling for the outcome of seropositive humans and livestock, included random effects (RE) on the district and nomadic camp level. For the human seroprevalence calculation, the clustering of individuals within nomadic camp was used as the cluster unit.

Univariable analyses of explanatory variables (biologically plausibly associated with brucellosis seropositivity) were evaluated by a logistic regression model with a RE at the nomadic camp level.

Seroprevalences of people and livestock species (also stratified to age classes) were calculated considering clustering within nomadic camps using Stata 10.5 (StataCorp LP, Texas, USA). A generalised linear latent and mixed model was used to assess the association between human and livestock seropositivity. This multilevel model allowed for inclusion of the denominator (number of people sampled per nomadic camp) in the analysis. Risk factors of human seropositivity, herd and individual livestock seropositivity analysis of associations, have been summarised in categories such as profession and reported symptoms. Factors possibly associated with seropositivity in humans and livestock (explanatory variables with *P* ≤ 0.2 in univariable analysis) were evaluated with multivariate logistic regression models (with RE at the nomadic camp level) using backward stepwise selection and a removal level for covariates at *P* = 0.10 based on the likelihood-ratio test (LRT).

The study was approved by the Ethics Committee of the Ministry of Health of Mongolia (ref. N 03/2010) and the Scientific Committee of Veterinary Research Institute (ref N. 256/2010). In Switzerland, approval was given by the Ethics Commission of the Cantons of Basel-Stadt and Basel-Land (Ethikkommission beider Basel) (ref. 169/10). Clinical brucellosis patients with a positive serological test result and clinical symptoms were registered and received doxycycline (100 mg) orally twice a day for 45 days and gentamicin (5 mg/kg) intravenously daily for 2 weeks. Informed written consent was obtained from all participants. In the case of children, informed consent was obtained from their parents or guardians.

## Results

### Seroprevalences in Humans and Animals

A total of 8054 livestock and dog sera and 574 human sera were tested with at least one serological test (Table 1). A total of 4123 livestock sera were collected from Sukhbaatar and 3931 livestock sera from Zavkhan province. The apparent human seroprevalence was 28.5% (95% CI 24.0–33.6) in Sukhbaatar and 25.9% (95% CI 20.2–32.6) in Zavkhan.

**Table 1:** Total sample size by species and number of samples examined with different diagnostic tests

Species	Total	RBT (India)^1^	RBT (Mongolia)^2^	RBT (Spain)^3^	ELISA IgG (human)^4^	ELISA IgM (human)^5^	ELISA (ruminant)^6^	ELISA (ruminant)^7^
Human	568	568			374	31		
Sheep	3338		1691	1647			242	131
Goats	3321		1988	1633			203	140
Cattle	817		366	451			130	55
Yaks	14			14				1
Camels	118		118				111	11
Horses	373		112	145			12	2
Dogs	72		72				46	13

^1^Rose Bengal Test produced by Tulip Diagnostic, India.

^2^Rose Bengal Test produced by Biocombinate, Mongolia.

^3^Rose Bengal Test produced by Brucellosis National Reference Centre, Spain.

^4^Indirect enzyme-linked immunosorbent assay detecting IgG in humans Diagnostic Automachine, USA.

^5^Indirect enzyme-linked immunosorbent assay detecting IgM in humans Nova Tech Immunodiagnostic GmbH.

^6^Competitive enzyme-linked immunosorbent assay detecting IgG in ruminants produced by VLA, UK.

^7^Competitive enzyme-linked immunosorbent assay detecting IgG in ruminants produced by Svanovir, Sweden.

In Sukhbaatar province, 318 herders and their family members (161 male and 157 female) participated from 83 nomadic camps. Participants were classified by their occupations as herders (*n* = 260), students/pupils (*n* = 45), children staying home (6), government employees (*n* = 4) and veterinarians (*n* = 3). Participants younger than 10 years had a seroprevalence of 16%. The seroprevalence was highest (>50%) among participants older than 45 years of age (Table 2).

**Table 2:** Human apparent seroprevalences by age class in Sukhbaatar and Zavkhan provinces

Age class	*n*	*n* pos^1^	% [95% CI binary outcome]	Seroprevalence^2^	95% CI^2^
Sukhbaatar					
<10 years	19	3	15.7 [3.4–39.6]	17.8	7.2–37.6
10–<15 years	17	1	5.9 [0.1–28.7]	6.1	0.8–32.7
15–<20 years	25	2	8.0 [0.1–26.0]	8.1	2.0–27.2
20–<45 years	184	48	26.1 [19.9–33.1]	26.1	20.3–32.8
≥45 years	73	37	50.7 [38.7–62.6]	50.9	38.6–63.1
Zavkhan					
<10 years	1	0	0	0	0
10–<15 years	10	3	30 [6.7–65]	na	na
15–<20 years	6	0	0	0	0
20–< 45 years	142	40	28.1 [20.9–36.3]	29	21.1–38.4
≥45 years	91	21	23.1 [14.9–33.1]	23.5	15.7–33.6

^1^Positive with the 1:1 RBT.

^2^Calculated with a random effect on the level of nomadic camp to consider potential clustering within nomadic camp.

In Zavkhan province, 256 herders and their family members (123 males and 133 females) participated from 69 nomadic camps, but only 250 blood samples were tested for serological tests. Participants were classified by their occupations as herders (*n* = 218), students/pupils (*n* = 16), government employees (*n* = 10), veterinarians (*n* = 7), children staying at home (*n* = 6) and disabled/unemployed (*n* = 5). Only 11 children younger than 15 years were sampled, but their seroprevalence was similarly high when compared with adults (Table 2).

Livestock seroprevalences were relatively homogenous between 5 and 8% in sheep, goats and cattle in Sukhbaatar province. In Zavkhan province, 2% of sheep and goats and 15% of cattle were seropositive. Seroprevalences were 3.4% in camels, 1% in horses and 41.3% in dogs in Sukhbaatar. In Zavkhan province, 11% of the tested horses were seropositive (Table 3). In Zavkhan province, only 14 yaks and 9 dogs were sampled.

**Table 3:** Apparent seroprevalence estimates of brucellosis in Sukhbaatar and Zavkhan province for humans and different livestock species in 2010

Species	*n*	Seroprevalence %^1^	95% CI
Sukhbaatar province			
Human	318	28.5	24.0–33.6
Sheep	1682	7.1	4.8–10.4
Goat	1671	5.1	3.0–8.6
Cattle	359	7.8	4.9–14.5
Camels	118	3.4	2.6–4.4
Horses	228	0.9	0.02–3.4
Dogs	65	41.3	29.38–54.36
Zavkhan province			
Human	250	25.9	20.2–32.6
Sheep	1656	1.9	0.9–3.6
Goat	1650	1.7	0.9–3.2
Cattle	458	15.3	9.9–22.7
Yak	14	0	
Horses	145	10.9	4.3–25.4
Dogs	9	0	

^1^Seroprevalence and 95% CI calculated with xtgee model specifying nomadic camp as a random effect.

The overall apparent seroprevalence of brucellosis was 27.3% in humans (95% CI 23.7–31.2%), 6.2% (95% CI 5.5–7.1%) in sheep, 5.2% (95% CI 4.4–5.9%) in goats, 16.0% (95% CI 13.7–18.7%) in cattle, 2.5% (95% CI 0.8–7.6%) in camels, 8.3 (95% CI 6.0–11.6%) in horses and 36.4% (95% CI 26.3–48.0%) in dogs. The overall apparent seroprevalence in yaks was not calculated because only 14 yaks were sampled during the study. In Table 4, true seroprevalences of humans, sheep, goats and cattle using the Rogan–Gladen formula show minor changes for humans and livestock.

**Table 4:** Comparison of apparent and true prevalences from Sukhbaatar and Zavkhan

Species	Apparent prevalence	Se	Sp	True prevalence
Sukhbaatar province				
Humans	28.5	0.92	0.91	28.2
Sheep	7.1	0.94	1	7.6
Goats	5.1	0.94	1	5.7
Cattle	7.8	0.997	0.99	6.9
Zavkhan province				
Humans	25.9	0.92	0.91	25.6
Sheep	1.9	0.94	1	2
Goats	1.7	0.94	1	1.8
Cattle	15.3	0.997	0.99	15.5

### Correlation Between Human and Livestock Seropositivity

Generalised linear latent and mixed models were run first in each species alone, then for three ruminant species all combined, and finally, regrouped as small ruminants and large animals (cattle, camel and horses). No association between the serostatus of the livestock and human status was found (data not shown).

Observations of livestock abortions were not associated with human brucellosis seroprevalence. The presence of a livestock species in a nomadic camp was not significantly associated with human seropositivity of any species. No livestock variable was significantly associated in the multivariable analysis, and thus was not considered in the risk factor analysis of human seropositivity (data not shown). Over 49% of participants (*n* = 556) reported that they fed aborted foetuses and placentas to the dogs.

### Reported Symptoms and Incidence of Apparent Brucellosis Seropositivity in Humans

Reported symptoms from disease events experienced during the previous month were compared to the sero-status of participants. Joint, muscle and back pain, weakness, night sweat, sleep disturbance and neuralgia were associated with seropositivity (Table 5). Night sweat and neuralgia remained in the stepwise backward multivariable model. In Sukhbaatar province, a total of 91 participants (*n* = 318) were seropositive, of which 62 (68%) reported at least one symptom and 54 (59%) at least two symptoms. Overall, there were 17% (95% CI 13–22%) of participants who had two cardinal symptoms of brucellosis at the time of interview. In Zavkhan province, among 64 seropositives, 41 (64%) have reported at least one symptom or a self-diagnosis and 34 (53%) reported two symptoms. Overall, there were 13.6% (95% CI 9.6–18.4%) of participants who were seropositive who had two cardinal symptoms of brucellosis at the time of interview. Among all seropositives in Sukhbaatar and Zavkhan, 66% had at least one symptom, whereas among the seronegative 47% had at least one symptom (*P* < 0.001; Table 5). Human incidence of apparent sero-conversion was estimated at 2.6% (95% CI 2.2–3.1%) per year in Sukhbaatar province and at 2.3% (95% CI 1.9–3.0) in Zavkhan province by Rose Bengal Test.

**Table 5:** Number of reported symptoms and human serostatus from Sukhbaatar and Zavkhan provinces

*N* reported symptoms	Seropositives		Seronegatives	
	*n*	%	*n*	%
Sukhbaatar				
0	29	32	101	44
1	14	15	41	18
2	6	7	22	10
3	8	9	13	6
>3	34	37	50	22
Zavkhan				
0	23	36	41	22
1	7	11	37	20
2	6	9	19	10
3	7	11	19	10
>3	21	33	70	38

### Risk Factors of Human Brucellosis Seropositivity

In Sukhbaatar, the univariable analysis showed significant associations with age classes (in comparison to adults 20–45 years, 10–20 year old participants were at lower risk, whereas the elders were at higher risk). Being a female was a risk factor, and students had increased risk when compared to herders. For behaviour related risk factors, the consumption of half-cooked liver alone was associated as a risk factor with seropositivity (Table 6). For Zavkhan, the same univariable analysis was carried out. Similar results were found for the association with age classes (Table 7). Older people had a higher risk and only the consumption of half-cooked liver was associated with being seropositive (Table 6).

**Table 6:** Univariable and multivariable analysis of risk factors of human seropositivity in Sukhbaatar province

Baseline	Univariable					LRT
	*n*	pos	%	OR	*P* value^1^	*P* (LRX2)
Districts						
Dariganga	89	26	28.6	1		0.24
Sukhbaatar	79	23	25.3	1	0.99	
Tuvshinshiree	77	16	17.6	0.6	0.21	
Khalzan	73	26	28.6	1.3	0.37	
Age (years)						
<10	19	3	15.8	0.5	0.34	<0.001
10 –<15	17	1	5.9	0.2	0.09	
15 –<20	25	2	8.0	0.2	0.06	
20 –<45	184	48	26.1	1	–	
≥45	73	37	50.7	3	0.001	
Sex						
Male	161	36	22.4	1	–	0.012
Female	157	55	35.0	1.9	0.013	
Occupation						
Herder	260	85	32.7	1	–	0.001
Student	45	3	6.7	0.15	0.002	
At home	6	1	16.7	0.4	0.42	
Other	7	2	28.6	0.8	0.82	
Raw milk consumption						
No	284	84	29.6	1		–
Yes	34	7	20.6	0.6	0.27	
Raw milk product consumption						
No	202	65	32.2	1		–
Yes	116	26	22.4	0.6	0.065	
Dried meat consumption						
No	205	60	29.3	1		–
Yes	113	31	27.4	0.9	0.73	
Raw liver consumption						
No	292	81	27.7	1		–
Yes	26	10	38.5	1.6	0.25	
Half-cooked liver consumption						
No	179	42	23.5	1		–
Yes	138	48	34.8	1.7	0.027	
Newborn						
No	122	36	29.5	1		–
Yes	195	55	28.2	0.9	0.80	
Direct contact to aborted foetus/retained placenta						
No	168	43	25.6	1		–
Yes	150	48	32	1.4	0.21	
Feeding dog abortions/placenta						
No	193	57	29.5	1		–
Yes	125	34	27.2	0.9	0.65	
Dumping of abortions/placenta						
No	283	80	28.3	1		
Yes	35	11	31.4	1.2	0.7	
Sheep abortions						
No	98	26	26.5	1		–
Yes	139	39	28.1	1.1	0.79	
Goat abortions						
No	93	21	22.6	1		–
Yes	145	45	31	1.5	0.16	
Cattle abortions						
No	217	62	28.6	1		–
Yes	20	3	15	0.4	0.20	
Mare abortions						
No	225	62	27.6	1		–
Yes	12	3	25	0.9	0.85	

^1^Level of significance is at *p* < 0.05

**Table 7:** Univariable analysis of risk factors of human seropositivity in Zavkhan province

Baseline	Univariable				
	*n*	pos	%	OR	*P* value^1^
District					
Durvuljin	96	18	19.6	1	
Ider	34	11	32.3	2.1	0.22
Ikh-Uul	88	24	27.2	1.6	0.30
Tosontsengel	38	11	30.5	2.0	0.22
Age (years)					
<10	1	0	0	–	–
10–<15	10	3	30	1.5	0.6
15–<20	6	0	0	–	–
20–<45	142	40	28.2	1	
≥45	91	21	23.1	0.8	0.41
Sex					
Male	120	36	30	1	
Female	130	28	22	0.6	0.18
Occupation					
Herder	218	55	25.4	1	–
Student	16	3	18.7	0.8	0.76
Other	22	6	27.3	1.1	0.94
Raw milk consumption					
No	234	61	26.0	1	
Yes	14	2	14.3	0.53	0.45
Raw milk product consumption					
No	118	30	25.4	1	
Yes	130	33	25.3	1.0	0.99
Dried meat consumption					
No	242	62	25.6	1	
Yes	5	0	0	1.4	1
Raw liver consumption					
No	246	62	25.8		
Yes	8	0	0		–
Half-cooked liver consumption					
No	124	32	25.8	1	
Yes	124	31	25.0	0.97	0.93
Newborn					
No	199	51	25.6	1	
Yes	47	12	25.5	1.1	0.77
Direct contact to aborted foetus/retained placenta					
No	102	25	24.5	1	
Yes	146	37	25.3	0.9	0.79
Feeding dog abortions/placenta					
No	146	36	24.6	1	
Yes	104	28	26.9	1.0	0.96
Dumping of abortions/placenta					
No	208	56	26.9	1	
Yes	42	8	19.0	0.55	0.251
Sheep abortions					
No	114	30	26.3	1	
Yes	109	28	25.6	1.1	0.79
Goat abortions					
No	155	40	25.8	1	
Yes	68	18	26.4	1.1	0.83
Cattle abortions					
No	178	48	26.9	1	
Yes	45	10	22.2	0.38	0.65
Mare abortions					
No	211	55	26.0	1	
Yes	12	3	25.0	0.88	0.87

^1^Level of significance is at *p* < 0.05

### History of Human Brucellosis Diagnosis and Treatment

In Sukhbaatar province, 18% of participants (*n* = 318) reported that they were diagnosed for brucellosis in the past. The median time of past brucellosis testing was 3.5 years (Q1 = 1.5 and Q3 = 20 years). Out of 14 participants diagnosed with brucellosis, 12 participants received treatment soon after the diagnosis made.

Seventy-nine nomadic camps reported that the nearest health centre was at the district and only seven nomadic camps were reported that the provincial hospital was the nearest. The median distance to the health centre was 20 km. The most frequent use of transport to reach the health centre was by motorcycle (84%), by car (27%), by horse or camel ride (12%), by walking (2%) and picked up by an ambulance of the health centre (1%).

In Zavkhan province, 15% of the participants (*n* = 254) reported that they had been diagnosed with brucellosis in the past. The median time of past brucellosis testing was 23 years (Q1 = 6.6 years and Q3 = 30.6 years). Among 21 participants (*n* = 38) diagnosed with brucellosis, 30% received brucellosis treatment. Among nine patients who received treatment, one patient was at a tertiary hospital, two were at a provincial hospital, four patients were at the district health centre, one patient went to the bag feldsher unit and one sought help elsewhere.

### Underreporting of Human Brucellosis

This study showed an apparent seroprevalence of humans of 26.0% in Zavkhan and 28.5% in Sukhbaatar provinces. In Sukhbaatar, 17 new human cases per 10,000 were reported in 2008 and 28 new cases per 10,000 in 2010 (Selenge et al. 2011). In Zavkhan province, there were no new human cases reported in 2008 and two new cases reported per 10,000 in 2010. An extrapolation of the results from Zavkhan to the whole country would mean that Mongolia had on average 6650 (95% CI 5180–8370) new brucellosis cases in 2010. This would represent an incidence of newly reported cases of 237 per 10,000 (95% CI 185–299) in 2010. Against the incidence of 15 humans per 10,000 reported in 2010, this represents a 15 fold underreporting. By using the data from Zavkhan rather than Sukhbaatar province, a more conservative estimate is calculated. Assuming that 50% of the seropositive have clinical symptoms (which was the case in our study) the incidence of clinical brucellosis in Sukhbaatar would be 131 (95% CI 110–154) per 10,000 and 119 (95% CI 93–150) per 10,000 population in Zavkhan. These results would indicate an underreporting of the annual incidence of clinical brucellosis by a factor of 4.6 (1307/280) in Sukhbaatar and by a factor of 59 (1188/20) in Zavkhan.

## Discussion

This is the first study of simultaneous assessment of brucellosis seroprevalence in humans and livestock. It shows a more complete epidemiological picture and deepens the understanding of infection patterns at the animal–human interface. The findings of this study are relevant for the country towards the development of a national brucellosis control programme by the medical and veterinary sectors. Our study provides evidence that brucellosis is a major public health problem among the rural population of Mongolia. All livestock species were seropositive by serological test. This means brucellosis may cause economic impact through a reduction of livestock productivity (Roth et al. 2003).

In the absence of a perfect test, brucellosis serology is difficult to interpret. All serological tests have limitations when used for screening (OIE 2009). In this study, the apparent seroprevalence has been adjusted for the performance of the Rose Bengal Test. However, it remains only an approximation of the true disease seroprevalence in the population. RBT and ELISA are suitable tests for screening at the national and local level according to the OIE guidelines. Two competitive ruminant ELISAs were used for livestock diagnosis and two different ELISAs were used for human diagnosis. More research is needed to identify the appropriate cut-off values for diagnostic tests in endemic settings. There are no specific serology tests for diagnosis in the horse, yak, camel and dog. We included horses, yaks and camels in the study because of the consumption of milk and milk products in Mongolia. Dog RBT results showed that dogs are likely to be infected. More research is needed to find appropriate serological tests and cut-off values for brucellosis diagnosis in yaks, camels, horses and dogs.

Traditional and cultural practices may influence zoonotic disease risk. Human seroprevalence was higher among women than men. Women play an important role taking care of newborn animals and milking all animals during the lactation period. Almost half of the participants reported that they fed dogs with aborted foetuses and placentas. Public education campaigns are needed among rural people informing them about the appropriate disposal of abortion by-products and other hygienic practices for preventing brucellosis.

### Access to Human Diagnosis and Treatment

According to the current national standard of human brucellosis diagnostics and treatments (MNS 5348-39:2003) any person with a positive result by standard RBT and history of exposure to infected livestock or consumption of dairy products should be considered as a suspected brucellosis case with a need to be confirmed by ELISA, PCR or bacteriology. Confirmed human cases receive brucellosis specific treatment requiring a 10 day hospitalisation, which is covered by the national insurance scheme. Outpatient treatments must be paid out of pocket. Unfortunately, most of the district health centres do not have any brucellosis diagnostic tests available and district medical doctors prescribe medical treatment based on clinical symptoms. Suspected brucellosis cases have to travel to the provincial hospital to receive a primary diagnosis by RBT. Provincial hospitals do not have ELISA or bacteriological methods to confirm the diagnosis. Therefore, suspected patients need to travel to the capital city to confirm a diagnosis. Brucellosis patients who are not diagnosed by a confirmatory test are not included in the official data registry, which may explain the high level of underreporting. The limited annual budget of district level health centres does not allow for covering 45 days of treatment cost for all patients.

Based on these findings, we recommended to the Ministry of Health (MoH) that standard RBT can be used as a primary test and modified RBT can be used as a confirmatory test at the district level. RBT is a cheap and simple test to perform at the district health centre under Mongolian conditions. Patients without complications can receive the 45 days of treatment regime as recommended by WHO without hospitalisation. These recommendations were accepted by the MoH, who modified the diagnostic method and accepted the proposed treatment regime. Currently, the MoH and NCCD are revising the national standards for human brucellosis diagnosis and treatment procedures according to our study results.

### Human Risk Factors

The higher risks for students compared to herders was difficult to interpret. The higher risk of women may be explained by their direct contact with weak newborns, feeding them at home and milking livestock during the lactation period. In contrast to a study in Kyrgyzstan (Bonfoh et al. 2012), we could not demonstrate a significant relationship between human and animal seroprevalence at the nomadic camp level. This may reflect complex contact networks which blur the detection of human-animal transmission at the nomadic camp level. In Zavkhan province, small ruminants had a higher seroprevalence than in Sukhbaatar aimag. This may be explained by differences in husbandry systems and local tradition. The milking of small ruminants is a tradition in Zavkhan province but not in Sukhbaatar province. More research is needed using molecular typing of circulating strains to ascertain the main transmission pathways in livestock and to humans.

The national brucellosis mass vaccination programme was officially implemented from 2000 to 2009. The government of Mongolia did not consider the livestock number when the mass vaccination budget was planned in 1999. The livestock numbers doubled during the intervention period but the number of vaccine doses was never adjusted for the livestock number since 2000 (Shabb et al. 2013). Based on structured questionnaires on livestock health information, 163 out of 169 nomadic camps reported that brucellosis vaccine was never offered to their herds by a local veterinarian in the last 3 years. Only one nomadic camp from Zavkhan province was included in the brucellosis vaccination programme in 2009. There was not enough vaccine, and there was a lack of monitoring of the vaccination coverage and quality control of the implementation of national vaccination programme at the village level. The vaccination coverage rate (35–53%) did not reach the critical immunization rate during the mass vaccination period from 2000 to 2003 year (Roth et al. 2003). The quality of the two brucellosis vaccines, in terms of viable colony forming units, and the cold chain were never assessed during the mass vaccination period. The poor performance of the past brucellosis mass vaccination campaign is certainly one of the reasons for the high seroprevalence of human brucellosis found in the study. Based on known vaccination coverage and the basic reproductive number, it can be ascertained that the transmission of brucellosis can be effectively interrupted (Zinsstag et al. 2005). If the coverage was found to be too low in some areas, then revaccination of those areas could be considered. Future mass vaccination campaigns must include an assessment of vaccination coverage for each of the animal species. The quality of the brucellosis vaccine and the cold chain should be followed up continuously. Independent vaccination coverage surveys help to monitor the effectiveness of the mass vaccination. Provincial level veterinarians and medical doctors have been trained on basic epidemiological concepts and methods implementing such coverage surveys for the ongoing mass vaccination campaign since 2011.

### Study Limitations

There are several limitations to this study. First, an immune-response proves exposure to *Brucella* (or cross-reacting bacteria) but not necessarily infection. Brucellosis serology is difficult to interpret due to the absence of perfect tests. The OIE guidelines for brucellosis state that RBT and ELISA are suitable screening tests at the local and national level. The RBT and ELISA should be validated at the country level using culture positive livestock sera and negative livestock sera from the national collection of reference sera. RBT and ELISA are currently not validated in Mongolia because of the lack of culture positive and negative sera. There may also have been temporal variations in pathogen exposure and clinical symptoms that were not captured by the cross-sectional study design.

## Conclusion

Our study reports a high seroprevalence of human brucellosis in Mongolia and indicates that the annually reported cases are significantly under-reported. Herders have limited access to brucellosis diagnosis at the district level which prevents them from receiving an adequate treatment. Human brucellosis can only be effectively controlled if livestock mass vaccination is implemented at high coverage. This is in compliance with the guidelines of the World Organization for Animal Health (OIE). The Mongolian government must implement effective monitoring of vaccination coverage and quality control of the implementation of mass vaccination programmes at the district and village level. Mass vaccination should be accompanied by public educational and communication programmes.

The study results influenced the public health policy of the MoH regarding the case definition and diagnosis of human brucellosis at the primary and secondary health care levels in the country. The MoH is currently revising the National Brucellosis Diagnosis and Treatment standard. The human and livestock linkage of brucellosis transmission was investigated by molecular typing of the *Brucella* spp. collected and is reported separately. The MoFA started implementing a vaccination coverage survey after the mass vaccination in 2012.
